# Easy-to-Use HPLC Method to Measure Intracellular Ascorbic Acid Levels in Human Peripheral Blood Mononuclear Cells and in Plasma

**DOI:** 10.3390/antiox11010134

**Published:** 2022-01-07

**Authors:** Gwendolyn van Gorkom, Birgit Gijsbers, Erik-Jan Ververs, Ahmed El Molla, Cindy Sarodnik, Celine Riess, Will Wodzig, Gerard Bos, Catharina Van Elssen

**Affiliations:** 1 Department of Internal Medicine, Division of Hematology, GROW School for Oncology and Developmental Biology, Maastricht University Medical Center, 6229 HX Maastricht, The Netherlands; b.gijsbers@maastrichtuniversity.nl (B.G.); ejververs@gmail.com (E.-J.V.); ahmedelmolla@outlook.com (A.E.M.); cindy.sarodnik@mumc.nl (C.S.); CelineRiess@gmx.de (C.R.); gerard.bos@mumc.nl (G.B.); janine.van.elssen@mumc.nl (C.V.E.); 2 Department of Clinical Chemistry, Central Diagnostic Laboratory, Maastricht University Medical Center, 6229 HX Maastricht, The Netherlands; will.wodzig@mumc.nl

**Keywords:** ascorbic acid, vitamin C, peripheral blood mononuclear cells

## Abstract

Given the growing interest in ascorbic acid (AA), there is a need for a reliable and reproducible method to measure AA status in the human body. Serum AA concentrations do not correlate well with tissue levels, but AA levels in leukocytes do. However, a standard method for clinical application is lacking. This present study describes a method to measure AA in the peripheral blood mononuclear cells (PBMCs) with hydrophilic interaction liquid chromatography (HILIC). The method can also be used in plasma and other leukocyte subsets. The measurements of AA in PBMCs and plasma were performed with HPLC with HILIC separation and UV detection. The sample preparation involved the isolation of PBMCs and lysis and precipitation with acetonitrile. European Medicine Agency guidelines for bioanalytic method validation were followed for the evaluation. A highly precise execution of the method was found with intra- and inter-assay variations at a maximum of 7.8%. In 40 healthy donors, a mean intracellular AA concentration of 7.9 microgram/10^8^ cells was found in PBMCs. A correlation between plasma and PBMC AA concentration was not present (r = 0.22). In conclusion, we developed a convenient, reliable, and reproducible method for the quantitative determination of AA within PBMCs and plasma from human blood.

## 1. Introduction

There is an increasing interest in the potential health benefits of vitamin C, or ascorbic acid (AA). AA is an essential vitamin that, unlike most other mammals, the human body cannot produce by itself, and has to be obtained from the diet [1]. Inside the human body, AA functions as an essential cofactor in numerous enzymatic reactions and as a potent antioxidant, due to its strong reducing potential [2]. For clinical purposes, AA concentration in serum or plasma is routinely determined by high-performance liquid chromatography (HPLC) in many laboratories globally and is an accepted golden standard for this measurement [3,4]. However, plasma and serum AA levels are very low compared with the rest of the human body and are highly affected by dietary intake, age, gender, and circadian rhythm. After oral intake of AA, the plasma AA increases rapidly, but this effect is only short-lived. These levels are thought to reflect the metabolic turnover rate of the vitamin [5,6]. Leukocytes can concentrate AA intracellular 80 times more than in plasma [2,5]. The leukocyte AA level is more stable than serum AA level because it is less affected by dietary changes and is also thought to be a more accurate assessment for AA storage [5]. Furthermore, AA is important for the development, proliferation, and function for different types of leukocytes [7,8]. Nevertheless, the measurement of intracellular leukocyte AA is hardly carried out because there is no well-established, standard, clinical method available.

For clinical investigators, the relationship between immune function and the AA concentration in leukocytes and plasma is highly relevant. Several methods for direct quantification of AA intracellular in leukocytes have been described with the use of the spectrophotometer (dinitrophenylhydrazine method) and reversed phase (RP) HPLC with UV or coulometric electrochemical detection [5,9,10]. With the described methods, however, estimates of the concentration of AA intracellular are quite variable, as AA degrades fast and it is not possible to use the same procedure for the measurement of AA in plasma. Therefore, we tried to adapt a standardized reverse phase (RP) HPLC method with UV detection for AA serum and plasma measurements in our routine clinical hospital laboratory to make it suitable for intracellular AA measurements. Sample preparation was optimized using several published methods of cell lysis, but they were either insufficient or interfered with the HPLC measurement. We left the standard method to measure AA in serum and developed a new method which, in comparison to previous published methods for PBMC AA detection, can measure AA both in PBMCs and plasma by hydrophilic interaction liquid chromatography (HILIC). In this article, we show that this method is effective, reliable, and reproducible. AA concentrations in separate cell fractions can also be measured with the same methodology, and only small amounts of cells are necessary. Furthermore, we show a very effective lysis process, which does not interfere with the measurement. 

## 2. Materials and Methods

### 2.1. HPLC and Conditions

The HPLC system used for the AA detection was as follows: HPLC liquid chromatograph pump, UV/VIS detector, column oven, auto sampler, degassing unit, and communications bus module (all Shimadzu) with HPLC column XBridge Amide 3.5 μm, 4.6 × 150 mm (Waters) and Guard column XBridge Amide 3.5 μm, 4.6 × 20 mm (Waters, Milford, MA, USA). The UV detection was at 255 nm wavelength. The mobile phase contained 85% acetonitrile and 15% water with 10 mM NH_4_Ac pH 7. The flow rate was 1 mL/min, the pressure 63 bar, the temperature of column oven 25 °C, the temperature of the auto sampler was 19 °C, and the injection volume was 20 μL. The concentration of vitamin C in the sample was specified using a calibration curve prepared beforehand using known concentrations of AA (Sigma Aldrich, Saint Louis, MO, USA) and D-(-) Isoascorbic acid (Sigma Aldrich, Saint Louis, MO, USA).

### 2.2. Sample Preparation

Lymphoprep density centrifugation was used to separate peripheral blood mononuclear cells (PBMCs) and plasma from heparin blood (600 g, 30 min). For enrichment of the different PBMC subsets (NK cells, T cells, B cells, and monocytes), MACs cell separation (Miltenyi Biotech, Bergisch Gladbach, Germany) with CD56, CD3, CD19, and CD14 microbeads was used (positive selection), according to the manufacturer’s protocol. In addition, cell sorting was performed on a BD FACS Melody to separate all different PBMC fractions.

Isolated cells were washed twice with PBS (centrifuged 283× *g*, 8 min) and erythrocytes were lysed when necessary, using lysis buffer. Lysis buffer was made with 200 mL Milli Q water plus 155 mM Ammonium chloride (NH_4_Cl, Merck), 10 mM potassium hydrogen carbonate (KHCO_3_, Merck) and 1 mM ethylene diamine tetra acetic acid (EDTA, Sigma Aldrich).

After cell count, 2 × 10^6^ cells were re-suspended in 100 μL 50 mM ammonium acetate followed by the addition of 400 μL precipitation agent. Precipitation reagent was made with 200 mL acetonitrile (Sigma Aldrich) plus 40 μL internal standard (D-iso ascorbic acid (Sigma Aldrich) stock solution 50 mg/mL in 0.1% metaphosphoric acid (Sigma Aldrich)).

After mixing thoroughly, the sample was incubated for 10 min in the dark at room temperature. To eliminate aggregated cell proteins, the procedure was followed by a final step of centrifugation (18.620 g, 10 min). Supernatant, containing the extracted AA, was then stored in brown vials at −80 °C until further analysis.

### 2.3. Method Validation

The validation was performed according to the European Medicine Agency’s (EMA) bioanalytical method validation guideline [11].

#### 2.3.1. Linearity and Quantification Limits

Linearity and quantitation limits were determined for ascorbic acid in plasma and in PBMCs. The limit of blank (LoB) was calculated using the formula: LoB = mean of blank + (1.645 × standard deviation (SD) of blank). Limit of detection (LoD) was calculated using the formula: LoD = LoB + (1.645 × SD of low concentration of AA). The lower limit of quantification (LLOQ) was determined by measuring serially diluted samples and determined to be the lowest value in which the coefficient of variation (CV) was under 10%. 

#### 2.3.2. Precision 

The intra-assay variation of the HPLC was investigated by measuring 4 quality control (QC) samples with different AA concentrations covering the range of the calibration curve (11µM, 20 µM, 35 µM, and 50 µM) 5 times in the same run and the inter-assay variation was calculated by measuring those 4 different concentrations 6 times on 2 different days.

The inter-assay variation of the isolation and extraction of intracellular AA from the PBMCs was investigated by performing the isolation, extraction, and analysis of different blood samples from a single individual 10 times.

#### 2.3.3. Accuracy

The results of plasma AA levels with our method were compared with those measured by a standardized method by Chromsystems, which is used in our hospital for clinical diagnostic purposes and that is validated with external controls provided by Instand every 3 months. Furthermore, accuracy for different concentrations was calculated by comparing the obtained concentration with the target concentration of the QC samples. 

Furthermore, the accuracy was assessed by comparing the results of QC samples of the intra-assay variation with the target concentration of AA.

#### 2.3.4. Sample Stability 

Samples were measured after different storage conditions. The first was isolated, lysed and measured immediately after the blood collection and served as reference point; the other blood samples were stored at room temperature and at 4 °C for 4 h and 72 h. Extracted samples were also measured fresh, frozen at −80 °C for a day, a week, and for 88 days.

#### 2.3.5. Carryover

Carryover was estimated by alternating injections of 10 samples with high (71 µM) and 11 with low (3 µM) concentrations of AA in a specified order according to an EP Evaluator^®^ (Data Innovations^®^, Colchester, VT, USA). Carryover results were compared to the error limit, which was defined as 3 times the standard deviation of the low after the low sample results.

### 2.4. Clinical Application

All blood specimens for validation were taken from healthy volunteers. The sampling was approved by the medical ethical committee which is in agreement with the Declaration of Helsinki and the volunteers signed informed consent. 

In order to gather local reference values, PBMC and plasma AA were determined in 40 healthy volunteers. None of these donors had any chronic diseases or were physically impaired. 

### 2.5. Calculations and Statistics

The concentration of vitamin C in every sample was calculated in µmol/L with a formula that was based on the calibration curve. The plasma AA level was identical to the concentration that was measured. From the concentration in the sample (100 µL), the intracellular amount of vitamin C in µg/10^8^ cells was calculated as intracellular vitamin C is most often displayed in these units (intracellular vitamin C (in µg/10^8^ cells) = measured concentration (in µmol/L) × 1.76). The concentration within the PBMCs was estimated based on the known average mean corporal volume of PBMCs (282.9 fL) [12].

Statistical analysis was performed with SPSS version 25. Results were expressed as mean, maximum, and minimum. A confidence interval of 95% was considered to indicate statistical significance. We used a Pearson’s correlation coefficient to investigate the association between PBMC AA level and serum AA level, and between serum and plasma AA level. For comparison of PBMC AA concentration in different genders and between leukocyte subgroups, an independent sample t-test was performed. 

## 3. Results

### 3.1. Sample Preparation 

Purity of the cells was established by flow cytometry and was found to be greater than 95%. After re-suspending the PBMCs in ammonium acetate, adding the precipitation reagent, and 10 min incubation, the lysis efficacy was evaluated by microscopy using Trypan blue. In a viable cell, Trypan blue is not absorbed; however, it traverses the membrane in dead cells. There were no intact lymphocytes visible, and thereby it was determined that the lysis method was efficient.

### 3.2. Method Validation

#### 3.2.1. Chromatography

**Figure 1:**
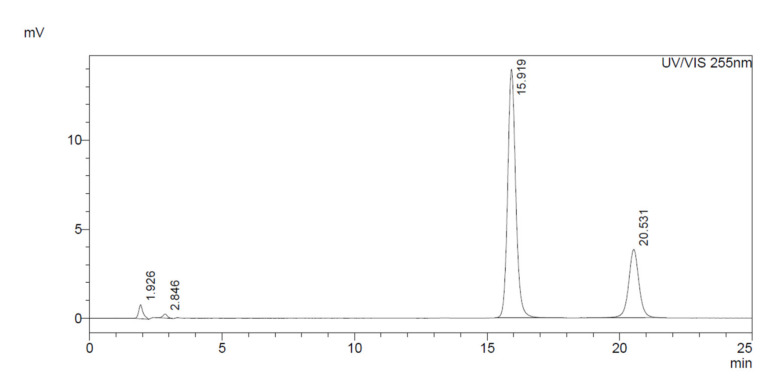
Chromatogram of a PBMC sample, in particular the vitamin C peak (at 20.5 min) and isoascorbic peak (IS) (at 15.9 min), spectrum taken as mass absorbance units.

The maximum AA peak was determined by performing a wave-length scan with AA in the mobile phase solution and showed a maximum peak at 255 nm. Using a flow rate of 1 mL/min, the AA and the internal standard peak were completely separated from other peaks and arrived at the detector after approximately 20.5 and 15.9 min, respectively (Figure 1). The peak authenticity was established by sample spiking. By running a sample with and without internal standard, it was shown that there were no underlying peaks at this timeframe in the sample. D-iso ascorbic acid was chosen as internal standard since it has almost the same properties as AA with regard to loss and decay, so even if the AA concentration in the sample is decreasing (for example while staying in the auto sampler of the HPLC), the ratio between internal standard and AA stayed equal after a maximum of 6 h.

#### 3.2.2. Linearity

**Figure 2:**
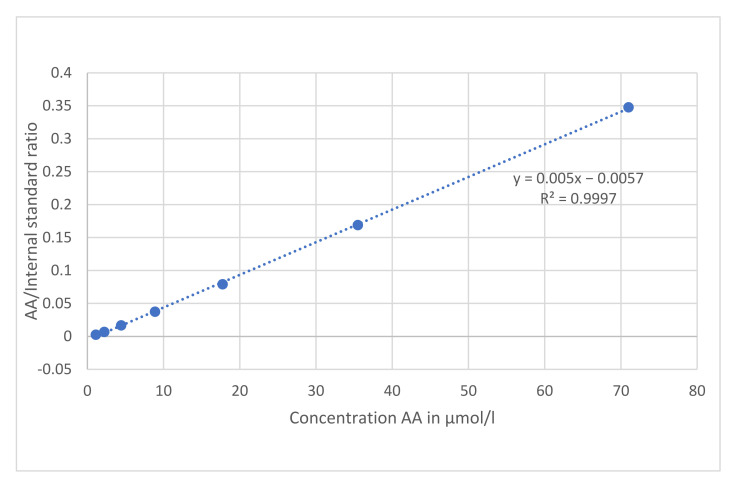
Example of a calibration curve.

The 7-point calibration graphs for ascorbic acid standards (0–71 µmol/liter) were plotted as the ratio of AA and internal standard showed linearity, passing though the origin (Figure 2): y = 0.005x − 0.0057, R^2^ = 0.9997.

#### 3.2.3. Quantification Limits

For the determination of the LoB, 9 blank samples were measured. LoB was calculated to be 1.65 µM (SD 0.26) and subsequently the calculated LoD was 1.93 µM (SD 0.17), when using repetitive measurements of a known sample of 1,1 µM from our standard curve. The lower limit of quantification (LLOQ) was determined by measuring serially diluted samples, with a concentration around the LoD. The deviation was >10% if there was less than 3.8 μM AA in the sample; thereby, the measurement is unreliable under this limit, according to our limit of 10%. From this, the calculated lower limit of quantification in the cells is 3.3 μg/10^8^ cells.

#### 3.2.4. Precision 

The intra-assay variation of the HPLC measurement was investigated by measuring 4 QC samples with different AA concentrations (11 µM, 20 µM, 35 µM, and 50 µM) 5 times, and was 0.6–7.7% (Table 1). The inter-assay variation of the HPLC measurement was calculated by measuring the samples with 4 different concentrations 6 times on 2 different days and was 0.9–2.5% (Table 1). Thereby, the total imprecision was 1.1–7.8%. Results gathered by this approach are therefore considered as reliable.

The inter-assay variation of the extraction of intracellular AA from the PBMCs was investigated by performing the isolation, extraction, and analysis of different blood samples from a single individual 10 times. The variation coefficient (CV) of the inter-assay variation of the isolation and extraction AA in the PBMCs was determined by performing the isolation, extraction, and analysis of separate blood samples of the same volunteer 10 times on the same day and was 7.7% (Table 2). 

#### 3.2.5. Accuracy

**Figure 3:**
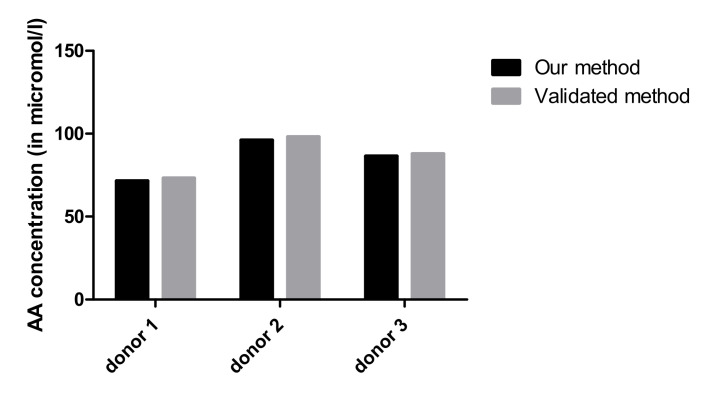
Comparison between plasma AA concentration with our method compared to the validated method.

The plasma AA levels determined with this method were compared with a validated method from Chromsystems used in our clinical diagnostic laboratory, and the results were similar (Figure 3). 

Furthermore, the obtained values of the QC samples of the intra- and inter-assay variation were compared with the nominal concentration (82–87% of the target value) of AA and the deviation was under 15% for all concentrations, except for the lowest concentration, which is acceptable, since it is within 20% of the nominal value (Table 3). 

#### 3.2.6. Stability

**Figure 4:**
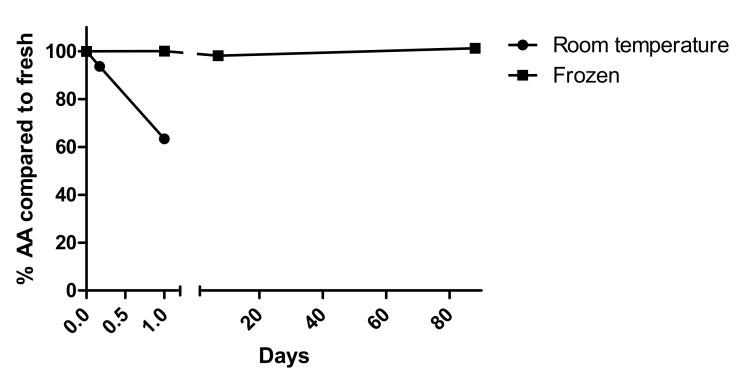
Stability of AA in blood sample stored at room temperature (mean of 3 samples) and of extracted AA in frozen at −80 °C (mean of 5 samples).

The AA concentration decreased by only 11% after the blood samples were stored for 4 h at room temperature in the dark, so we consider this as acceptable (Figure 4). After 24 h storage at room temperature, the AA levels decreased on average 38%. Storage at 4 °C was even less stable. The freezing process of prepared samples did not influence AA levels, and after 1, 7, and 88 days of storage at −80 °C, there was less than 5% change in repeated measurements (Figure 4).

#### 3.2.7. Carryover 

Carryover was measured by subtracting the mean of low–low results (low values that follow a low value, 3.03 µM) from high–low results (low values that follow a high value, 3.17 µM) of 10 replicates of a sample with high (71 µM) AA concentration, and 11 replicates of a low (3 µM) AA concentration (Supplementary Table S1). There was no significant carryover, since the measured carryover (0.13) was lower than the error limit (0.40).

### 3.3. Reference Values

**Figure 5:**
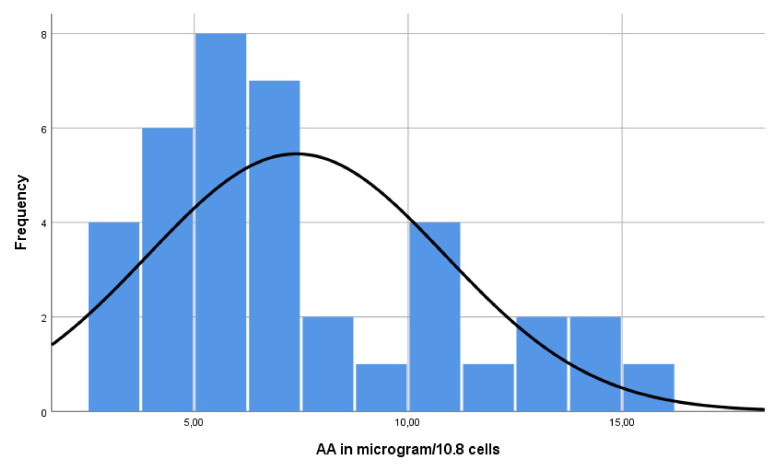
Distribution of PBMC A concentration in healthy volunteers.

In order to gather local reference values, PBMC and plasma AA were determined in 40 healthy volunteers. These consisted of 11 males and 29 females with a mean age of 35 years (range 18 to 62). The mean of the PBMC AA concentration was 7.9 µg/10^8^ cells (range 3.17 to 27.47). There was no Gaussian distribution of the intracellular AA concentrations with a skewness of 2.16 and a kurtosis of 6.76 (Figure 5). On average, the PBMCs contained 24 times higher vitamin C concentrations than plasma (1586 μM vs. 67 μM). No differences based on age or gender were found in this population.

**Figure 6:**
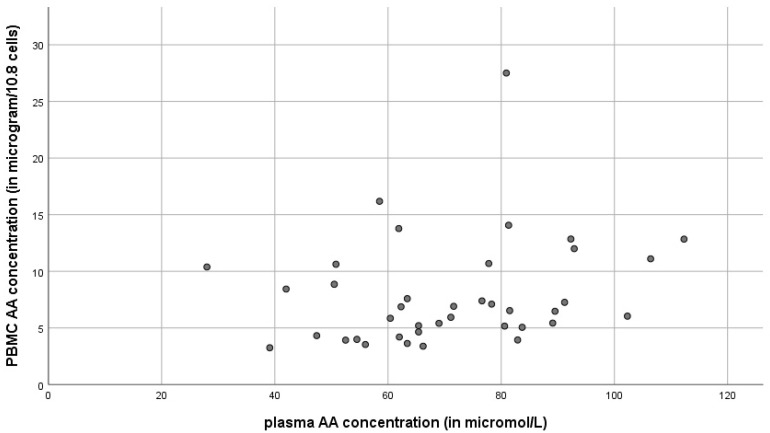
Correlation between the plasma ascorbic acid concentration and the PBMC ascorbic acid concentration, *n* = 40, r = 0.22.

Data on plasma and PBMC AA concentrations are represented in Figure 6. There was no correlation (r = 0.22) between plasma and PBMC AA levels. Even though the values were not always equal, plasma AA levels had a strong positive correlation with serum AA levels, that are normally used in clinical applications (r = 0.70, *p* < 0.01) (data not shown). 

### 3.4. PBMC Subsets

**Figure 7:**
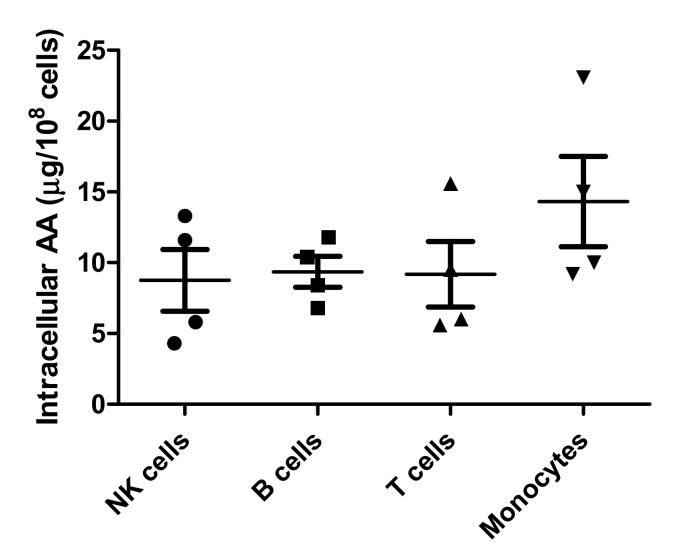
Amount of AA in µg/10^8^ cells for each subset (*n* = 4).

To determine if there was a difference in AA content in different PBMC subtypes, we measured AA levels in PBMC subsets (T cells, B cells, NK cells, and monocytes) in 4 healthy volunteers. In 3, we isolated the cells using MACS separation, and in 1 we used cell sorting by flow cytometry. Results are given in Figure 7. The AA levels in different subgroups were not significantly different (*p* = 0.32). They were also similar with the different separation methods. 

## 4. Discussion

In this article, we describe an optimized method for quantitative determination of AA in human PBMCs and in plasma using HPLC with HILIC mode. HPLC is already considered as a golden standard for the measurement of AA in serum, but a standard clinical method to measure it intracellular in PBMCs was not available. At first, we tried to measure intracellular AA using a standardized method for serum AA determination, but we did not succeed to use this HPLC protocol due to interfering signals after lysis. After that, we chose HILIC mode, as it works well for small, very polar molecules, such as AA, and it is well studied for the detection of AA in other circumstances [13]. 

In most articles about intracellular AA measurements in leukocytes, the method is not validated according to the European Medicine Agency guidelines for bioanalytic method validation. In one article, in which the validation is clearly described, RP-HPLC was used and information about lysis efficacy was lacking [10]. We tried to use sonication for lysis based on this article, but the efficacy was very low in our hands (30–40%). If the lysis is that inconsistent, AA concentrations intracellularly will be underestimated. Our lysis procedure was very efficient. Furthermore, plasma AA levels measured with our method were almost identical to plasma AA levels determined with the standardized method used for clinical applications.

We analyzed 40 blood samples of healthy adult volunteers. These results showed a mean AA concentration of 7.9 µg/10^8^ cells in PBMCs. In other studies, there is a large variety in these results (5.3 to 122 µg/10^8^ cells) [5,10,14,15,16]. The values we found are in the same range as earlier findings. One study, in which AA levels of 20 healthy volunteers were measured by HLPC with coulometric electrochemical detection, shows identical results (5.3 to 10.6 µg/10^8^ cells) [14]. In other studies, the detected amount of AA in PBMCs was higher, but in these studies the measured samples were possibly contaminated with platelets, that are known to have intracellular AA as well [5,17,18]. In one of the more recent papers, AA is measured in lymphocytes using RP HPLC with UV detection and a mean AA of 23.5 µg/10^8^ cells was found [10]. However, these measurements were done in blood samples of children that already needed blood examination; therefore, these measurements might not represent healthy control values, especially in adults [10]. In our reference population, the calculated intracellular concentration of the PBMCs was around 1.6 mM. This is also in line with earlier findings of Levine et al. who describe the effects of AA supplementation and deprivation on intracellular values of lymphocytes and monocytes in 7 healthy volunteers [6]. 

In the literature, more is known about the amount of AA that can be found in leukocytes and in most articles and textbooks reference ranges of 20 to 53 µg/10^8^ cells are given [9,18,19,20]. However, in most of these analyses, the buffy layer is used, containing not only leukocytes but also platelets and, thereby, again, overestimating the AA found in the leukocytes [18]. The relationship between buffy layer and leukocyte AA levels was researched by Gibson et al. and it was determined that with a normal amount of platelets, the buffy layer concentration shall be divided by 2.0, but this conversion factor shifts if the number of platelets is abnormal [17]. We checked the amount of platelets that were left in our cell samples by flow cytometry and found that the percentages of thrombocytes in the samples were quite low (median 1% of total cell count, whilst in the buffy layer with normal cell counts the leukocyte:platelet ratio is around 1:40). These small amounts of platelets will have an irrelevant influence on the total AA concentration measured with this method as it is thought that platelets only carry 0.25–0.55 µg AA/10^8^ cells [5,17].

Our reference values have some limitations, for instance, underrepresentation of higher ages. They are probably regional and are influenced by lifestyle and diet. Samples were taken in summer; values could be different in other seasons. For clinical use of this method, it is thereby advised to create local reference values. Similar to data from other studies, AA concentration in lymphocytes was not well correlated with plasma concentration in healthy volunteers. 

We also used the same method to investigate AA levels in different leukocyte subsets and noticed no significant differences. This shows that determination in even small subsets of the peripheral blood leukocytes is possible since only 2 × 10^6^ cells are necessary for a reliable result with this method. 

In our clinical setting, in an earlier investigation, we observed low serum AA levels in patients after stem cell transplantation, a patient group in which the immune system functioned less, which is very vulnerable to infections [21]. This is interesting, as we discovered before that in vitro lymphocytes and NK cells are dependent on AA for proper development and/or proliferation [22,23]. For an accurate estimation of the relevance of AA in relation to the function of the PBMCs in these kinds of clinical situations it is interesting to measure intracellular AA levels in the PBMCs, or even in subgroups. Moreover, our method can be used for in vitro studies on the function of leukocytes in correlation to intracellular AA levels where we have little knowledge on how optimal intracellular concentrations should be in relation to the function of these cells. 

## 5. Conclusions

We developed an effective and reliable method for the quantitative determination of ascorbic acid in human PBMCs by HPLC in HILIC mode, for which you only need a small amount of cells and that can also be used for measurement in plasma and in different leukocyte subgroups. Intracellular AA values found with this method are in line with earlier results in the literature. The presented methodology can be of use in a large variety of patients, as it can possibly better determine if there is a true deficiency in the total body AA. Therefore, this measurement could be added to AA measurements in plasma in many laboratories. Furthermore, AA seems relevant in many diseases, but a clinical effect of supplementation is not always present in intervention studies. However, in these studies, the effect of AA supplementation is measured in plasma, which is not a proper reflection of total AA. This measurement can be useful in these intervention studies to determine the optimal dose and administration route of AA supplementation and to identify patients with a true AA deficiency. 

## Figures and Tables

**Table 1:** Precision test with intra- and inter-assay variation for AA measurement with HPLC. Target (µM) is the required concentration of the quality control samples (QC1, QC2, QC3, QC4). SD is the standard deviation. CV is the ratio of the standard deviation to the mean. TI is the total imprecision.

Ascorbic Acid	QC1		QC2		QC3		QC4	
	Intra-Run	Inter-Run	Intra-Run	Inter-Run	Intra-Run	Inter-Run	Intra-Run	Inter-Run
Target (µM)	11		20		35		50	
Number	5	6	5	6	5	6	5	6
Mean (µM)	9.5	9.0	17.1	17.0	30.0	29.9	43.1	43.4
SD	0.73	0.1	0.19	0.2	0.27	0.7	0.25	0.7
CV (%)	7.7	1.1	1.1	1.0	0.9	2.5	0.6	1.6
TI (%)	7.8		1.1		2.7		1.7	

**Table 2:** Inter-assay variation of the intracellular measurement of the PBMCs.

Extraction Number	PBMC AA (in µg/10^8^ Cells)
1	11.31
2	11.79
3	9.80
4	11.16
5	9.31
6	10.75
7	10.61
8	10.97
9	9.71
10	9.89
MEAN	10.53
SD	0.81
CV (%)	7.72

**Table 3:** Accuracy for AA measurement with HPLC. Target concentration (in µM) is the required concentration of the quality control samples (QC1, QC2, QC3, QC4).

Ascorbic Acid	QC1		QC2		QC3		QC4	
	Intra-Run	Inter-Run	Intra-Run	Inter-Run	Intra-Run	Inter-Run	Intra-Run	Inter-Run
Target (µM)	11		20		35		50	
Number	5	6	5	6	5	6	5	6
Mean (µM)	9.5	9.0	17.1	17.0	30.0	29.9	43.1	43.4
Accuracy (%)	86	82	86	85	86	85	86	87
Deviation (%)	14	18	14	15	14	15	14	13

## Data Availability

Data is contained within the article and supplementary materials.

## Supplementary Materials

The following supporting information can be downloaded at: https://www.mdpi.com/article/10.3390/antiox11010134/s1, Table S1: Carry over determination.
