# Supplementary material for: Easy-to-Use HPLC Method to Measure Intracellular Ascorbic Acid Levels in Human Peripheral Blood Mononuclear Cells and in Plasma
Source: Antioxidants (Basel). 2022 Jan 7;11(1):134. doi: 10.3390/antiox11010134 (PMC8773372; doi:10.3390/antiox11010134)
Supplement: Supplementary file 1 [file antioxidants-11-00134-s001.zip › antioxidants-1543066-supplementary.pdf]

antioxidants

MDPI

Article

Easy-to-use HPLC method to measure ascorbic acid levels intracellular in human peripheral blood mononuclear cells as well as in plasma

Gwendolyn van Gorkom[1], Birgit L.M.G. Gijsbers[1], Erik-Jan Ververs[1], Ahmed El Molla[1], Cindy Sarodnik[1], Celine Riess[1], Will Wodzig[2], Gerard Bos[1], Catharina Van Elssen[1]

[1] Department of Internal Medicine, Division of Hematology, GROW School for Oncology and Developmental Biology, Maastricht University Medical Center, Maastricht, The Netherlands
[2] Department of Clinical Chemistry, Central Diagnostic Laboratory, Maastricht University Medical Center, Maastricht, the Netherlands
* Correspondence: gwendolyn.van.gorkom@mumc.nl;

Table S1. Carry over determination. Low-Low: Low concentration sample measured following a low concentration. High-Low: Low concentration sample measured following a high concentration sample. SD: Standard deviation.

Sample	Results (µM)
Concentration low	3.0
Concentration high	71.0
High-Low mean	3.17
Low-Low mean	3.03
Low-Low SD	0.13
Error limit	0.40
